# Biocompatibility and Antibacterial Properties of ZnO-Incorporated Anodic Oxide Coatings on TiZrNb Alloy

**DOI:** 10.3390/nano10122401

**Published:** 2020-11-30

**Authors:** Oleksandr Oleshko, Yevheniia Husak, Viktoriia Korniienko, Roman Pshenychnyi, Yuliia Varava, Oksana Kalinkevich, Marcin Pisarek, Karlis Grundsteins, Oksana Pogorielova, Oleg Mishchenko, Wojciech Simka, Roman Viter, Maksym Pogorielov

**Affiliations:** 1 Medical Institute, Sumy State University, 40018 Sumy, Ukraine; oleshkosanya007@gmail.com (O.O.); evgenia.husak@gmail.com (Y.H.); vicorn77g@gmail.com (V.K.); pshenychnyi@gmail.com (R.P.); yuliia.varava@gmail.com (Y.V.); o.s.pogorielova@gmail.com (O.P.); 2 Institute of Applied Physics NASU, 40000 Sumy, Ukraine; oksana.kalinkevich@gmail.com; 3 Institute of Physical Chemistry PAS, 01-224 Warsaw, Poland; mpisarek@ichf.edu.pl; 4 Institute of Atomic Physics and Spectroscopy, University of Latvia, LV-1586 Riga, Latvia; karlis.grundsteins@gmail.com; 5 NanoPrime, 39-200 Dębica, Poland; dr.mischenko@icloud.com; 6 Faculty of Chemistry, Silesian University of Technology, 44-100 Gliwice, Poland

**Keywords:** ZnO nanoparticles, TiZrNb alloy, plasma electrolytic oxidation, cell culture, antibacterial properties

## Abstract

In a present paper, we demonstrate novel approach to form ceramic coatings with incorporated ZnO nanoparticles (NPs) on low modulus TiZrNb alloy with enhanced biocompatibility and antibacterial parameters. Plasma Electrolytic Oxidation (PEO) was used to integrate ZnO nanoparticles (average size 12–27 nm), mixed with Ca(H_2_PO_2_)_2_ aqueous solution into low modulus TiZrNb alloy surface. The TiZrNb alloys with integrated ZnO NPs successfully showed higher surface porosity and contact angle. XPS investigations showed presence of Ca ions and absence of phosphate ions in the PEO modified layer, what explains higher values of contact angle. Cell culture experiment (U2OS type) confirmed that the surface of as formed oxide-ZnO NPs demonstrated hydrophobic properties, what can affect primary cell attachment. Further investigations showed that Ca ions in the PEO coating stimulated proliferative activity of attached cells, resulting in competitive adhesion between cells and bacteria in clinical situation. Thus, high contact angle and integrated ZnO NPs prevent bacterial adhesion and considerably enhance the antibacterial property of TiZrNb alloys. A new anodic oxide coating with ZnO NPs could be successfully used for modification of low modulus alloys to decrease post-implantation complications.

## 1. Introduction

Dental and orthopedic implants fulfil to specific requirements, such as high strength, low density, excellent corrosion resistance, high biocompatibility, and ability to osteointegration. Elastic stiffness is an important parameter that predicts load transfer between implant and surrounding bones. Most implants are still made of Ti alloys with elastic module (Young modulus) above 100 GPa. Their load transfer could lead to osteoporosis due to mechanical mismatch [1]. The implants with both high strength and a low Young’s modulus (close to bone values—16–20 GPa) are required for dental applications [2]. It has been demonstrated that decreasing of elastic modulus minimized the bone atrophy due to reduction of stress shielding bone resorption and prevented implant loosening [3,4]. Metastable beta Ti-based alloys are the best candidates for dental and orthopedic implant development due to superelastic effect (high recoverable strain) and a very significant reduction of the apparent elastic modulus [5]. Beta-titanium alloys such as metastable TiNb exhibit significantly low apparent elastic modulus and demonstrate promising results on in-vitro and in-vivo research [6]. Maity et al. developed Ti-35Nb-7Zr-5Ta alloy with low Young’s modulus (E~68 GPa) and high flow strength (σf ~1 GPa) with uniform hardness distribution without any noticeable change in the strength of the material [5]. Nune et al. reported on high bioactivity of Ti-24Nb-4Zr-8Sn alloy (~49 GPa), including ability of osteoblast’s stimulation [7]. Previously, we developed and investigated new TiZrNb system with extremely low Young’s module equal 34.85 GPa with high biocompatibility [8].

Mechanical properties of implants play important role in a long-term exploitation period. It requires active surface for successful osteointegration in early post-implantation time-point. The implant surface parameters such as chemical composition and roughness provide significant influence to protein adsorption, precipitation of bone minerals, and stimulation of cells growth after implantation [9]. Calcium-phosphate coatings demonstrate significant improvement of osteointegration due to direct osteoblast stimulation and collagen adhesion [10]. However, current deposition methods show short-term stability and frequent implant failures [11]. Additionally to osteointegrative potential, implant surface should provide protection from bacteria adhesion and biofilm formation for prevention of infectious complications [12]. Recently, metal and metal oxide nanoparticles are actively used to provide antibacterial properties of implant calcium-phosphate coating [13,14,15,16]. Silver is the most known metal with high antibacterial properties that demonstrates significant reduction of bacteria adhesion in numerous researches [13,14]. But side-effects, including cell toxicity and tissue accumulation [15,16] require development of new strategy to balance antibacterial properties and osteointegration potentials. Zinc and zinc oxide are promising candidates due to significant antibacterial and osteogenic properties [17,18,19]. Zn exhibits promotion of specific osteoblast gen markers and stimulates mineralization via the deposition of calcium in mesenchymal stem cells [17]. Some reports suggested that Zn nanoparticles (ZnNPs)-decorated implant surface demonstrate increasing initial cell adhesion, spreading and proliferation [18,19]. At the same time, ZnNPs demonstrate moderate antibacterial properties due to direct cell-NPs interaction and Reactive Oxygen Species (ROS) evolution [20]. Recently, Ag/ZnO-hydroxyapatite coating deposited by laser cladding demonstrated prevention of bacteria adhesion and stimulation of the new bone tissues formation in-vivo [17]. Other study demonstrated that ZnO/polydopamine (PDA)/arginine-glycine-aspartic acid-cysteine coatings exhibit both antibacterial and osteogenic potential [21]. Authors confirmed that controlled release of Zn ions from coating could be an ideal solution for balancing osteogenic/antibacterial strategy.

Plasma Electrolytic Oxidation (PEO) is a quite new promising method for formation of stable ceramic coatings containing calcium phosphates on titanium and its alloys [22]. The main advantage of the PEO process is formation of good quality coatings on samples with complex shapes. The coatings are composed of substrate oxides and incorporated compounds from electrolyte [23,24]. It is possible to incorporate insoluble compounds/particles directly into the coating from electrolyte [25]. In our previous papers we proved fabrication of bioactive and antibacterial oxide coatings on Ti or ZrNb alloy with incorporation of nanoparticles [26,27,28]. PEO process provides formation of stable coating resistant to corrosion with slow ion release [26].

In a present paper, we demonstrate novel approach to form anodic ceramic coatings with incorporated ZnO NPs on low modulus TiZrNb alloy. The obtained coatings show both antibacterial and osteogenic effect.

## 2. Materials and Methods

### 2.1. Chemicals

The TiZrNb alloy (NanoPrime (Dębica, Poland) with a height of 6 mm and 6 mm diameter was used in experiment [8]. All chemicals for ZnO NPs synthesis and PEO process (Zn(CH_3_COO)_2_∙2H_2_O, Ethylene glycol, Ca(H_2_PO_2_)_2_) have been purchased from Sigma Aldrich (Darmstadt, Germany) and used as received. *Staphylococcus aureus* and *Esherihia coli*, obtained from the Bacteria Collection of Sumy State University (Sumy, Ukraine), were used in bacteriological experiment. All bacteriological media were taken from HiMedia (Maharashtra, India) and Alamar blue was from Invitrogen (Carlsbad, CA, USA). For the cell culture study, all media and reagents were purchased from Gibco^®^, Gaithersburg, MD, USA.

### 2.2. Synthesis and Characteerization of ZnO Nanoparticles

Nanoparticles were synthesized by polyol synthesis. Ethylene glycol (EG) was used as the reaction medium. 2.195 g of Zn(CH_3_COO)_2_∙2H_2_O salt was dissolved in 10 mL of EG in a three-necked flask. A thermocouple, connected to a heating mantle with magnetic stirring, was placed in the flask with a reflux condenser.

The resulting solution was heated with vigorous stirring with a Teflon stirrer. After reaching a temperature of 160 °C, the mixture was kept for 60 min. Nanostructured zinc oxide was formed as a result of the decomposition reaction of the zinc acetate:

Zn(CH_3_COO)_2_∙2H_2_O → ZnO + 2CH_3_COOH + H_2_O

**Scheme 1:**
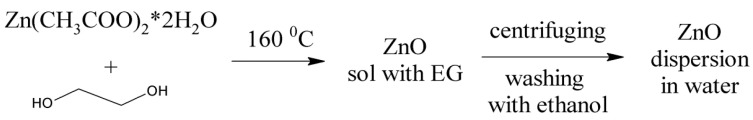
The ZnO nanoparticle synthesis process.

The mixture was cooled to room temperature and the synthesized product was separated from the organic component (EG) by centrifugation. EG residues were washed with ethanol with vigorous shaking followed by centrifugation. The washing operation was repeated three times. Schematically, the process of obtaining ZnO can be represented by the following Scheme 1:

### 2.3. Plasma Electrolytic Oxidation (PEO)

The surface area of the modified TiZrNb alloy was equal to 0.28 cm^2^. The samples were anodized in Ca(H_2_PO_2_)_2_ (0.5 M) electrolyte without and with ZnO nanoparticles (40 g/L). PEO of the samples surface was realized via DC galvanostatic anodization (anodic current density = 150 mA/cm^2^) using a DC power supply (PWR 800H, Kikusui, Japan) at a limiting voltage of 300–500 V for 5 min [26]. The samples were marked as TiZr-X for Ca(H_2_PO_2_)_2_ electrolyte and TiZr-X-ZnO for electrolyte with ZnO NPs, where X is the limiting voltage.

### 2.4. Surface Analysis

Scanning electron microscopy (SEM), energy-dispersive X-ray (EDX), X-ray photoelectron spectroscopy, and contact angle (CA) methods were used for structural and surface characterization of the modified surface. Details of these experiments were detailed described in our previous paper [26].

To investigate a thickness of the coatings formed during the PEO process, the cross-sections analysis tests of the oxide layers were performed. The samples were embedded in epoxy resin (Eposir F 740 + Ipox EH 2260) and then placed in a vacuum unit for 3 min before drying overnight. The grinding machine (Einhell TH-US 400) with 1440 rpm was used to prepare cross-sections by using abrasive paper (Hermes BW114) with 400, 1000, 1200, and 1500 granulation. SEM images of cross-sections were obtained by using scanning electron microscopy (SEO-SEM Inspect S50-B (FEI, Brno, Czech Republic; accelerating voltage—15 kV). To assess the chemical composition of the PEO layers, an energy-dispersive X-ray spectrometer (AZtecOne with X-MaxN20, Oxford Instruments plc, Abingdon, UK) accompanied by SEM was used.

Optical properties of non-treated and PEO coated TiZrNb samples were studied by photoluminescence spectroscopy in the range of 350–800 nm. Ultraviolet Nd:YAG laser (266 nm, output power 27 mW, CNIlaser, Changchun, China) was used for photoluminescence excitation. HR4000 Ocean Optic spectrometer (Orlando, FL, USA) was used for detection of photoluminescence.

### 2.5. Antibacterial Assessment

The *Staphylococcus aureus* (*S. aureus*, B 918) obtained from the National Collection of Microorganisms (D.K. Zabolotny Institute of Microbiology and Virology, Kyiv, Ukraine) was cultivated on nutrient agar for 24 h. The suspensions of overnight strain were mixed with nutrient broth medium, and the final concentration of microorganisms was adjusted to 10^6^ CFU using a McFarland standard.

The minimum inhibitory concentration (MIC) and minimum bactericidal concentration (MBC) were evaluated before PEO deposition of ZnO-NPs by using the serial dilution method. ZnO-NPs (initial concentration 3 g/L) were diluted in each well of sterile 24-well plastic plate to have dilutions 1:2:4:8 etc. Then, 100 µL of bacteria suspension was added to each well to reach 10^5^ CFU/mL. The test and control samples (bacterium suspension alone) were incubated under shaking conditions (Mini Rocker-Shaker, BioSan MR-1, Riga, Latvia) at 37 °C for 24 h. Afterwards, 10 µL of suspension from the wells with no visible bacterial growth (MIC) were cultivated on Mueller–Hinton’s agar plates at 37 °C for 24 h to evaluate MBC.

Antibacterial parameters of ZrTiNb PEO coated discs assessed with time-dependent adhesion test. For this purpose, samples were immersed horizontally in 2 mL bacterial suspension with a concentration 10^5^ CFU/mL and statically incubated for 2, 4, 6, and 24 h aerobically at 37 °C. At each time point, the disks were washed three times with phosphate buffer saline (PBS; pH = 7.4) to remove medium and planktonic bacterial cells. Then samples were placed individually in tubes with sterile saline and were ultrasonicated for 1 min in an ultrasonic bath (B3500S-MT, Bransone Ultrasonics Co., Shanghai, China) followed by vortex mixing (Mini Rocker-Shaker, BioSan MR-1, Riga, Latvia). 10 µL aliquots of each sonicated liquid were pipetted out and spread onto Mueller–Hinton’s agar plates using the streak plate technique. The plates were incubated at 37 °C for 24 h. The number of adhering viable microorganisms was assessed by counting the colonies. All the measurements were triplicated.

### 2.6. Cell Culture

The cells (U2OS cell type) were grown in 75 cm^2^ cell culture flasks under standard culture conditions of humidified air containing 5% CO_2_ at temperature 37 °C with medium renewal every 2–3 days. Dulbecco’s modified Eagle medium/nutrient mixture F-12 (DMEM/F-12) with L-glutamine was used, containing 100 units/mL penicillin, 100 µg/mL streptomycin, 2.5 µg/mL amphotericin B and 10% fetal bovine serum. Experiments were performed as described before [26]. After sterilization samples were placed in a separate well of 24-well cell culture plate and immersed in DMEM overnight. U2OS cells were seeded on each sample and in the wells without samples (as positive control) with the cell density of 10⁴ cells per well. Cell adhesion and proliferation on samples were assessed by the Alamar blue colorimetric assay as following: the plates were incubated for 8 h at 37 °C in the dark and then medium was added. In next step, medium was transferred to another 96-well plate, and the absorbance was measured by using a Multiskan FC (Thermo Fisher Scientific, Waltham, MA, USA) plate reader at wavelengths of 570 and 595 nm. The cells were quantified at different time intervals: 1, 3, and 7 d. All experiments were repeated three times.

All experiments with cell culture were approved by Institutional Bioethics Committee (Sumy State University, protocol 5/25 from 8 May 2019).

The statistical analysis of obtained results was based on one-way analysis of variance (GraphPad Prism 8.0 software), and *p* value of less than 0.05 was considered to be statistically significant and detailed described in [26].

## 3. Results and Discussion

### 3.1. ZnO NPs Characterization

**Figure 1:**
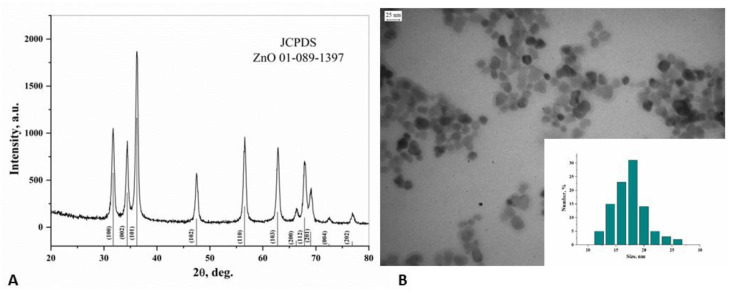
Diffraction pattern (**A**) and TEM image (**B**) size distribution (insertion) of ZnO nanocrystals.

The results of XRD analysis of the synthesized sample dried at 80 °C for 12 h showed that well-crystallized zinc oxide of the hexagonal modification (space group P63mc) was formed (Figure 1A). The characteristics of the diffraction peaks of the synthesized ZnO correspond to the JCPDS card No. 01-089-1397.

The average size of ZnO was calculated from the physical broadening of the diffraction peak (102) using the equation:

$$L=\frac{0.94\lambda }{\beta cos\;\theta \;},$$

where *λ* is the x-ray diffraction wavelength; *β* is the value of the physical broadening of the corresponding diffraction peak; *θ*—diffraction angle. Crystalline size of ZnO was 13.6 ± 5 nm.

The calculated unit cell parameters of the synthesized zinc oxide were *a* = 0.32476 nm, *c* = 0.52095 nm, and the value of elementary cell volume was *V*_cell_ = 0.0549 nm^3^.

Analysis of TEM microscopy images showed that the synthesized nanoparticles had a spherical shape with sizes in the range of 12–27 nm (Figure 1B).

ZnO nanoparticles have shown their effectiveness against *S. aureus* strain (MIC and MBC were 0.375 g/L and 0.1875 g/L, respectively).

### 3.2. Surface Analysis of PEO Ceramic Coatings

**Figure 2:**
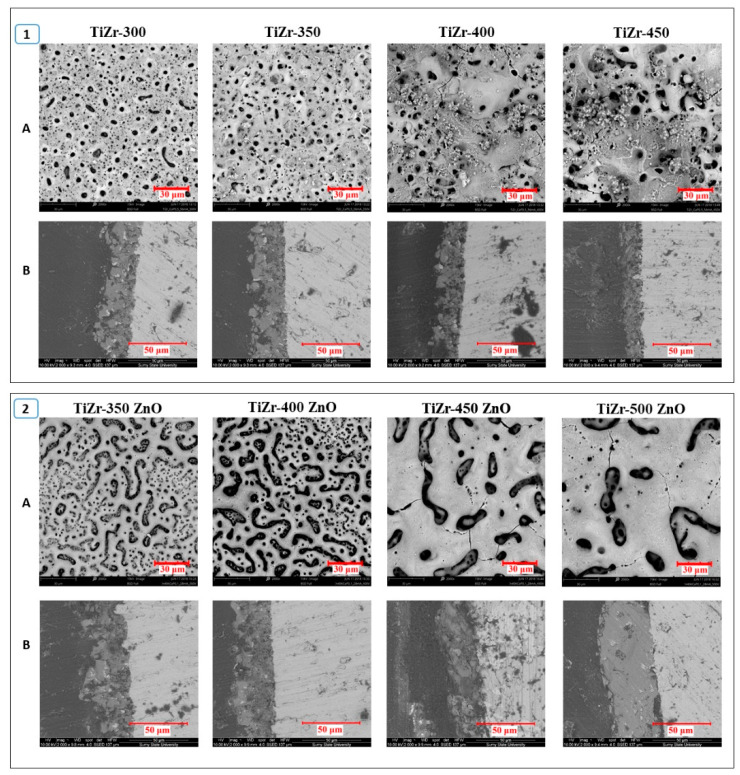
The SEM images microscopy of oxide layer (**A**) and cross-section (**B**) of Plasma Electrolytic Oxidation (PEO)-treated implants in Ca(H_2_PO_2_)_2_ electrolyte bath (**1**) and with ZnO nanoparticles (NPs) (**2**).

**Figure 3:**
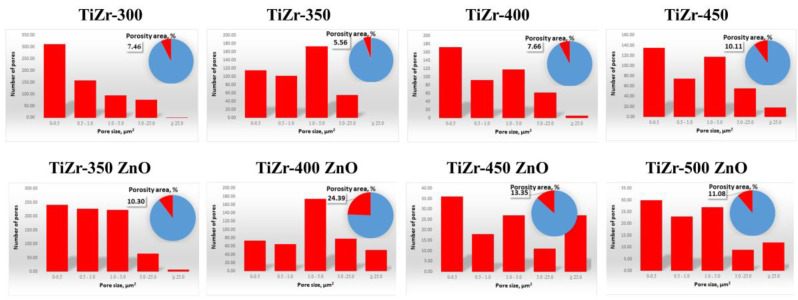
The pore size distribution of PEO-treated implants in Ca(H_2_PO_2_)_2_ electrolyte bath (**upper row**) and with ZnO NPs (**low row**).

Scanning electron microscopy demonstrates formation of a thick homogeneous oxide layer on TiZrNb samples after PEO in Ca(H_2_PO_2_)_2_ electrolyte bath (Figure 2). The thickness of PEO coating significantly decreased from 29.7 ± 6.3 µm at 300 V mode to 14.85 ± 5.8 µm at 450 V (Figure 2). The surface was covered by an oxide layer, containing small round-shaped pores and craters with various sizes. Figure 2 and Figure 3 demonstrate that surface structure, pore growth and pore size distribution strongly depend on PEO regimens (voltage applied). The surface porosity significantly grew from 7.46 ± 0.54% to 10.11 ± 1.2 with increasing voltage. The addition of ZnO NPs leads to significant growth of oxide layer thickness from 37.8 ± 5.3 µm at 350 V to 45.9 ± 3.7 µm at 500 V regime. Surface morphology after PEO with ZnO NPs is shown in Figure 2. Irregular elongated large pores with numerous round-shaped small pores have been identified (Figure 2). The increase of voltage led to decreasing of small pores number and increasing of elongated pore size (Figure 3). Opposite to basic Ca(H_2_PO_2_)_2_ electrolyte, addition of ZnO NPs led to dramatically increasing porosity from 10.30 ± 2.4% at 350 V mode to 24.39 ± 6.2% at 400 V followed by porosity reduction to 13.35 ± 3.9% and 11.08 ± 4.3% at higher voltages. Pore size and porosity were the main parameters responsible to cell attachment and proliferation. It was found that increasing of pore size significantly facilitate bone cell migration and proliferation [29,30] as well as cell biochemical activity (ALP and collagen production) [31]. On the other hand, the mesoporous surface could provide more natural environment for cell attachment and proliferation, and ZnO NPs solution provides formation of hierarchical architecture surface on TiZrNb alloy.

EDX analysis showed that increasing voltage up to 400 V had no effect to amount of titanium, zirconium, calcium and phosphorus content in the oxide layer formed in Ca(H_2_PO_2_)_2_ electrolyte (Table 1). Decrease of concentration of titanium, calcium and phosphorus and increase of concentration of zirconium to 3.5% was observed at higher voltages. Atomic ratio of Ca/P increased from 1.1 to 1.3, similarly to the Ca/P atomic ratio in hydroxyapatite [32]. Incorporation of ZnO nanoparticles to the implant surface affected the concentrations of elements (Ca, P, Zr, Ti, Zn) (Table 1). The ZnO content was 7% for low voltages (350–400 V) and 10% for high voltages (450–500 V) The Ca/P ratio was independent of voltage—0.4–0.6. Niobium was not detected in all cases due to low concentration in the oxide layers.

**Figure 4:**
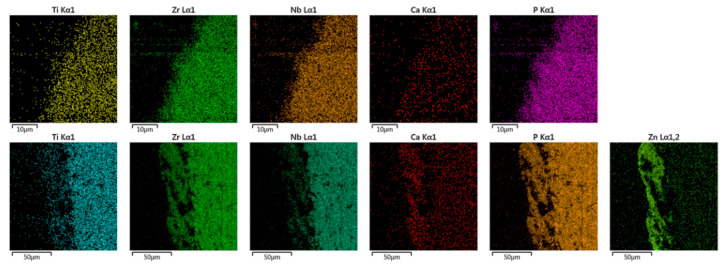
Exemplary EDX mapping of TiZrNb alloy cross-sections after PEO in Ca(H_2_PO_2_)_2_ electrolyte without (**upper row**) and with addition of ZnO-NPs (**low row**).

Figure 4 shows distribution of elements in the oxide layers formed in Ca(H_2_PO_2_)_2_ electrolyte (upper row) and after addition of ZnO NPs (low row). We can confirm uniform distribution of Ca, P, and Zn (in the case of second electrolyte) in whole volume of the coatings.

**Figure 5:**
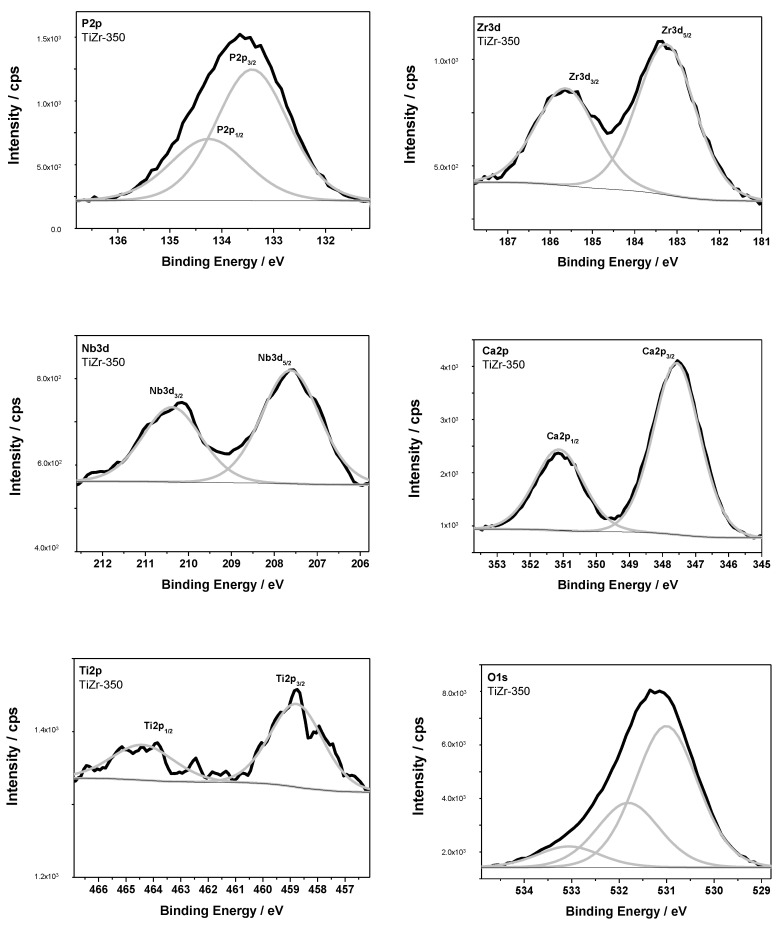
The high resolution XPS spectra after the deconvolution procedure, sample TiZr-350.

**Figure 6:**
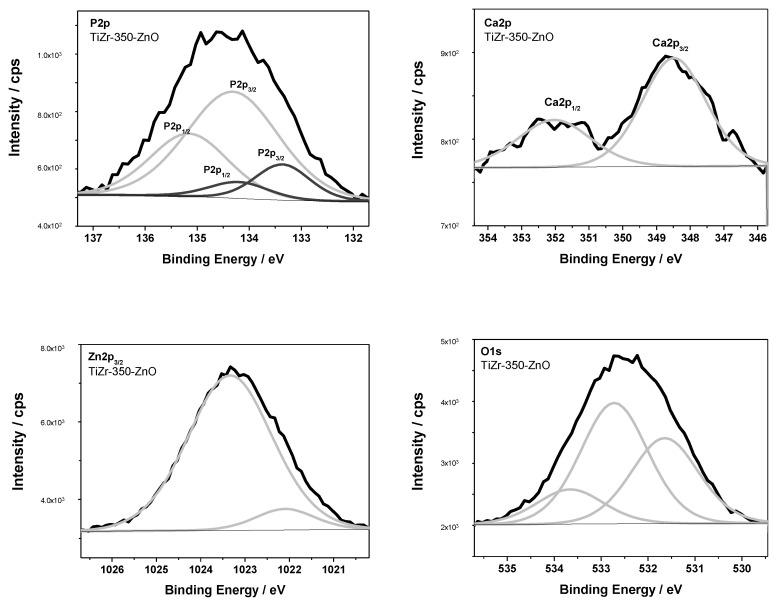
The high resolution XPS spectra after the deconvolution procedure, sample TiZr-350-ZnO.

The PEO coatings formed on the TiZrNb alloy were analyzed by using an XPS technique. XPS results are summarized in Figure 5 and Figure 6 and Table 2.

Table 2 shows the binding energies of Ti2p3/2, Zr3d5/2, Nb3d5/2, Zn2p3/2, P2p3/2, Ca2p3/2 and O1s signals registered at the surface of TiZr-350 and TiZr-350-ZnO samples after deconvolution procedure. The measured XPS spectra for metallic elements were characteristic for oxides. Therefore, it was found that the signals at each individual binding energies could be attributed to titanium oxide (458.8 eV—TiO_2_), zirconium oxide (183.3 eV—ZrO_2_) and niobium oxide (207.6 eV—Nb_2_O_5_) for sample without the addition of ZnO (Figure 5). Whereas, these oxides were not detected on the surface of TiZr-350-ZnO sample (Figure 6).

Binding energies of zinc oxide (ZnO—1022.1 eV) and (1023.3 eV) were observed. For the TiZr-350 sample, the high resolution XPS spectra of the P2p, Ca2p exhibited dominant peaks located at 133.4 eV and 347.6, corresponding to calcium phosphate functional groups. The peak at 531.9 eV for the O1s spectrum came from the PO_4_^3−^ lattice. In the case of the samples with incorporated ZnO, characteristic binding energies were also observed for phosphate groups (133.4 eV—P2p3/2, 531.6 eV—O1s) and also for metaphosphate groups (134.3 eV—P2p3/2, 532.7 eV—O1s) of a predominant nature. We can suppose that phosphorus compounds in the presence of ZnO precursors were preferentially formed during layer formation. Therefore, no typical binding energies, characteristic for calcium phosphates were detected on the sample surface. Only the presence of Ca–Cl bonds was found. These results correlated well with EDX analysis. Presence of Cl could be connected with the ZnO nanoparticle synthesis procedure.

**Figure 7:**
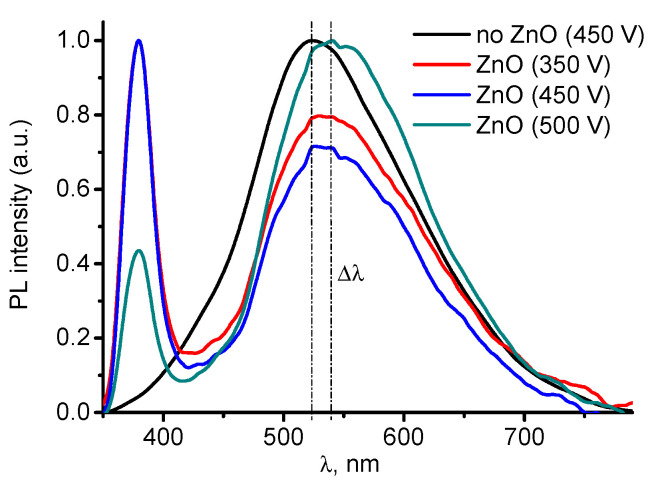
Normalized photoluminescence spectra of ZnO-incorporated oxide coatings on TiZrNb alloy.

Normalized photoluminescence (PL) spectra of ZnO-incorporated oxide coatings are shown in Figure 7. PEO treated samples without ZnO incorportion showed broad asymmetric peak, centered at 524 nm. The observed peak corresponded to TiO_2_ emission [33]. The observed emission was related to oxygen vacancies and self-trapped excitons [34]. After the PEO process with ZnO nanoparticles the emission spectra changed. Red shift of emission peak from 524 to 530–536 nm was observed. New peak at 379 nm appeared in ZnO-incorporated oxide coatings. It is known that ZnO has two emission bands: UV (375–384 nm), related to excitons, and visible (430–700 nm), related to structure defects [35]. The changes of PL spectra after ZnO incorporation showed the formed ZnO nanostructures on the sample’s surface. For instance, new UV peek at 379 nm corresponded to exciton emission of ZnO. Red shift of visible emission pointed to photoluminescence from ZnO defects (oxygen vacancies) [36]. The ratio between UV and visible emission pointed to crystallinity and defect concentrations in the ZnO. This ratio decreased with increase of limited voltage in the PEO process. According to EDX Zn concentration increased with enhancement of applied voltage. However, increase of the PEO voltage stimulated forming of defects in ZnO, which might influence other properties, such as contact angle and biocompatibility.

**Figure 8:**
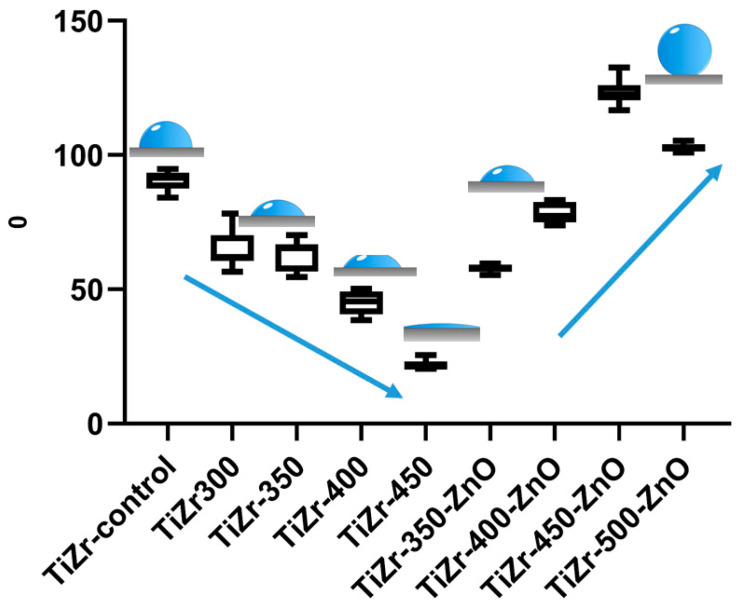
Contact angle distribution of TiZr alloys after treatment in different solution. Blue arrow demonstrates trends in Ca(H_2_PO_2_)_2_ electrolyte bath and with ZnO NPs.

Contact angle (CA) is an essential parameter of medical implant surface due to interaction with biological fluids. The hydrophilic surface is favorable for cell attachment but in the same case for bacteria colonization. And vice-versa—pure bacteria attachment to a hydrophobic surface is accompanied by low cytocompatibility [37]. The CA of non-treated TiZr surface was 93.6 ± 4.90 and significantly decreased after PEO, up to 21.1 ± 3.70 at 450 V (Figure 8). High wettability may play a significant role in biocompatibility during implantation due to fast surface protein absorption after blood-implant contact. The addition of ZnO NPs to electrolyte caused CA shift to hydrophobic values—from 58.2 ± 6.30 at 350 V to 123.1 ± 14.20 at 450 V. Antifouling properties of hydrophobic surface could induce additional antibacterial effects. On other hand, it could decrease cell attachment to the surface.

### 3.3. Cell Culture

**Figure 9:**
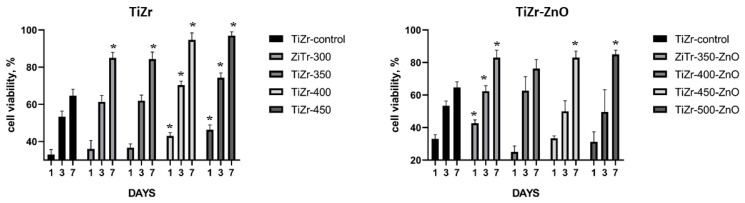
Resazurin reduction assay of U2OS cells cultured on TiZr substrate after PEO in Ca(H_2_PO_2_)_2_ bath and with ZnO NPs. (*—significant difference (*p* ≤ 0.05) between PEO coated samples and polished control).

Titanium and its alloys demonstrated high biocompatibility and ability to support cell proliferation [5,7,17]. Our experiment showed appropriate cell adhesion on day 1 for non-modified TiZrNb alloy surface with next cell proliferation—the viability rate rich 66.8 ± 4.7% at day 7 (Figure 9). Ceramic coatings formed at 400 and 450 V in Ca(H_2_PO_2_)_2_ electrolyte demonstrated significantly higher cell attachment at first day. This relates to low contact angle providing higher level of protein absorption from culture media. As result, the enhancement of cell attachment capacity for modified surface was observed. All surfaces obtained in the PEO process demonstrated significantly higher cell proliferation compared the non-treated control group (Figure 9). As previously reported, the present in the PEO coatings calcium and phosphorus are osteoinductive agents [32,33]. The sample surface, modified with ZnO NPs did not demonstrate significant difference in cell attachment compared to the control samples, except for the TiZr-350-ZnO one, that was characterized by low contact angle. Only at the 7th day was significant cell proliferation found. However, the cell viability rate was still below the level obtained for samples anodized in the Ca(H_2_PO_2_)_2_ electrolyte. We confirm that ZnO NPs-contained ceramic oxide coating demonstrated a hydrophobic nature that could affect primary cell attachment. Ca–P coating influenced proliferative activity of attached cells stimulating competitive adsorption of cells and bacteria in clinical situation.

### 3.4. Bacterial Adhesion Test

**Figure 10:**
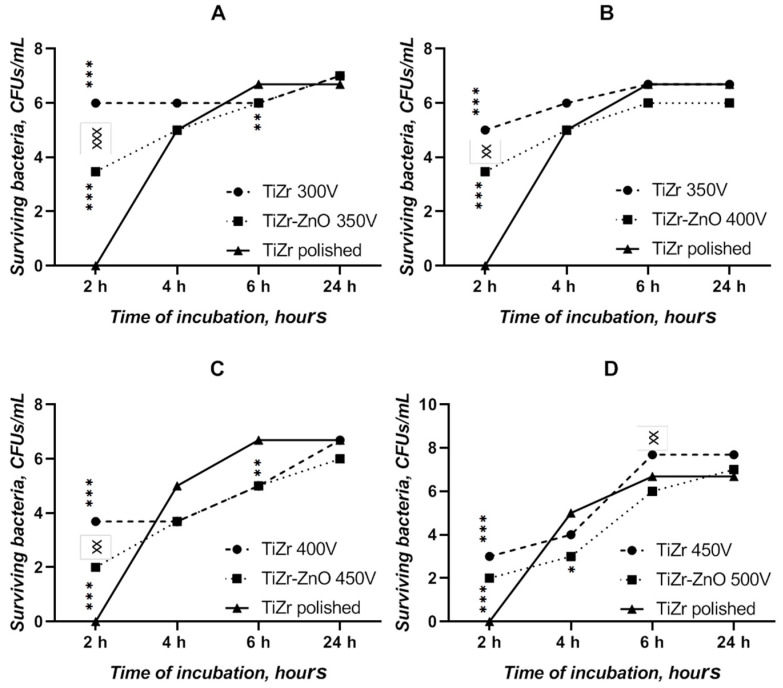
Comparison of the time-dependent adhesion effect of TiZr and TiZr-ZnO samples (**A**)—samples TiZr-300-ZnO and TiZr-350-ZnO, (**B**)—TiZr-350-ZnO and TiZr-400-ZnO, (**C**)—TiZr-400-ZnO and TiZr-450-ZnO, and (**D**)−TiZr-450-ZnO and TiZr-500-ZnO. *−significant difference (*p* ≤ 0.05) between PEO coated samples and polished control (**−*p* ≤ 0.01 and ***−*p* ≤ 0.001), x—significant difference (*p* ≤ 0.05) between TiZr and TiZr-ZnO samples.

Figure 10 represents that samples with a ceramic oxide layer exhibited higher adhesive activity in comparison to polished control after 2 h co-cultivation with bacterial suspension. There was no significant difference between TiZr-X and TiZr-X-ZnO samples almost for all subsequent contact times. Nevertheless, all TiZr-X-ZnO samples (except those obtained at 500 V), showed significantly higher antibacterial activity after 2 h of incubation. On the other hand, the TiZr-500-ZnO sample exhibited a noticeably higher antibacterial effect against on *S. aureus* in 6 h of co-cultivation. It can be observed that both types of PEO-treated samples had higher antibacterial activity (*p* < 0.005) in comparison to the polished disks at this time point. The most significant activity was revealed for all ZnO NP-containing samples; otherwise, better antibacterial performance was demonstrated by TiZr-450-ZnO and TiZr-500-ZnO ones.

The antibacterial properties of ZnO NPs was realized through the Reactive Oxygen Species (ROS) mechanism [20] and Zn ions release that leads to bacteria cell membrane penetration with following cell apoptosis [38]. However, high roughness of PEO coated samples induces accelerated adhesion of bacteria [21]. Other possible mechanism that prevent bacteria adhesion is high contact angle that exhibit antifouling machanism [39]. Hence, alloys with a higher level of contact angles exhibited more substantial antibacterial properties against the studied bacteria. They reduced bacteria concentration on their surfaces, preventing biofilm formation.

The revealed antibacterial characteristics of both types of sample surfaces (TiZr-X and TiZr-X-ZnO samples) are under the influence of the surface nanotopography. The present work suggests that ZnO NPs may be acceptable for PEO treatment solutions because they did not facilitate bacterial adhesion and considerably developed the antibacterial property of TiZrNb alloys. That is vital for the prevention of chronic inflammatory responses caused by commensal microorganisms as they can stimulate inflammatory cytokines and chemokines, leading to tolerance and active immunity disbalance [40].

## 4. Conclusions

Plasma electrolytic oxidation of TiZrNb alloys in a Ca(H_2_PO_2_)_2_ electrolyte with and without ZnO NPs formed a stable ceramic coating. Oxide layer of ceramic coatings formed in the electrolyte without ZnO NPs was composed of substrate oxides with incorporated Ca and P elements, in a form of calcium phosphate. Addition of ZnO NPs to the electrolyte changed chemical composition of the coating with reduction of Ca and P concentration and formation of metaphosphates. However a new ZnO NP loaded surface provided appropriate environment for osteoblast cell adhesion and exhibited medium grade antibacterial activity.

## Figures and Tables

**Table 1:** The semi-quantitative EDX analysis of PEO coatings.

	Atomic Concentration, %						Ca/P Ratio
	O	Ca	P	Ti	Zr	Zn	
TiZr-300	68.6	14.7	13.3	1.8	1.6	-	1.1
TiZr-350	68.7	14.8	13.6	1.5	1.5	-	1.1
TiZr-400	70.0	14.2	13.2	1.2	1.4	-	1.1
TiZr-450	71.7	13.3	10.4	1.2	3.5	-	1.3
TiZr-350 ZnO	68.6	4.3	9.9	3.7	7.1	6.5	0.4
TiZr-400 ZnO	70.1	3.9	9.5	3.2	6.8	6.5	0.4
TiZr-450 ZnO	67.6	5.4	9.4	2.1	5.8	9.7	0.6
TiZr-500 ZnO	68.2	5.2	9.8	2.1	4.7	9.9	0.5

**Table 2:** The XPS data evaluated from the deconvolution of Ti2p, Zr3d, Nb3d, Zn2p, P2p, Ca2p, O1s XPS spectra recorded for the TiZr-350 and TiZr-350-ZnO samples.

Samples	Binding Energy (BE)/eV							Chemical Bonds
	Ti2p_3/2_	Zr3d_5/2_	Nb3d_5/2_	Zn2p_3/2_	P2p_3/2_	Ca2p_3/2_	O1s	
TiZr-350	458.8						531.1	Ti-O (TiO_2_)
		183.3					531.1	Zr-O (ZrO_2_)
			207.6				531.1	Nb-O (Nb_2_O_5_)
					133.4	347.6	531.9	calcium phophates
TiZr-350-ZnO				1022.1				Zn-O (ZnO)
				1023.3				ZnO precursor
					133.4		531.6	phosphate [PO_4_]^3−^, Zn-O
					134.3		532.7	metaphosphate [PO_3_]^−^
						348.5		Ca-Cl (CaCl_2_)
